# What Happened if Various Kinds of Postconditioning Working on the Preconditioned Ischemic Skin Flaps

**DOI:** 10.1371/journal.pone.0072818

**Published:** 2013-09-12

**Authors:** Lin Huang

**Affiliations:** Plastic and Reconstructive Surgery, Anzheng Hospital, Capital University of Medical Science, Beijing, People’s Republic of China; Medical University Innsbruck, Austria

## Abstract

**Objective:** Ischemic pre-conditioning and post-conditioning are useful manipulations to reduce the undesirable effects of ischemia-reperfusion skin flap each. But the impact of post-conditioning on the pre-conditioning skin flap is not manifested. Here we investigated the influence of ischemic post-conditioning in a preconditioned axial pattern skin flap model.

**Method:** We used the skin flap in 40 rabbits and divided them into 5 groups randomly. At first we induced the ischemic pre-conditioning of the flap which was applied by 2 periods of 15 minutes of ischemia/15 minutes of reperfusion cycle. Next post-conditioning was performed by 6 cycles of 10 seconds of repeated ischemia/reperfusion periods at different times of just after the reperfusion,5 minutes after the reperfusion,10 minutes after the reperfusion. The animals were allocated into 5 groups: group 1 (Ischemia Group); group 2: (Pre-conditioning Group); group 3: (Pre-conditioning+ Post-conditioning Group); group 4 (Pre-conditioning+ Post-conditioning 5 minutes later Group); group5 (Pre-conditioning+ Post-conditioning 10 minutes later). The neutrophil count was assessed with histologic analysis before the dissection of the skin flap. Flap viability was assessed 1 week after the operation, and surviving flap area was recorded as a percentage of the whole flap area. LSD test was used for statistical analysis among different groups to evaluate the effects of ischemic pre-conditioning against ischemia.

**Result:** Among the varying groups, the neutrophil count varied: Group 1 was50.12±5.91; Group 2, 30.00±2.00, and Group 3, 18.87±3; Group 4, 22.50±1.92; Group 5, 30.12±1.88.The mean± SD surviving areas of the flaps for groups 1, 2, 3, 4 and 5 were 31.76±4.59, 51.26±3.24,82.18±5.28,66.85±3.87 and 51.13±2.90 respectively. Spearman correlation analysis shows an increase relation between neutrophil count and flap survival rate in the different groups (P <0.05).

**Conclusion:** Ischemic post-conditioning has protective effect on ischemic preconditioned skin flaps, but the post-conditioning should be performed within 5 minutes after the end of ischemia.

## Introduction

Advances in reconstructive surgery have led to an increasing use of extensive skin flaps for the closure of surgical defects. Free skin flaps are widely used for this purpose. But ischemia-reperfusion injury is underlying (partial) flap necrosis. Now a new concept has emerged during the past years, which consists of pre-conditioning or post-conditioning the tissue by exposing it to a sub-lethal degree of environmental stress prior to surgery [1–6].

During the relieving of the reperfusion injury of the free skin flap, the role of the ischemic pre-conditioning on the skin flap has been proved by Tatlidede S. In our previous studies we demonstrated a beneficial effect of tissue post-conditioning on the survival of critically ischemic free skin flaps in new zeweland rabbits. After the ischemia postconditiong of the skin flap, the mean ±SD surviving flap portion improved greatly which was 31.64±1.04% compared to 48.95±0.82% [7]. But whether the ischemic post-conditioning could influence the effect of pre-conditioning skin flap or not has not been confirmed.

Here we hypothesize the additional of post-conditioning of the skin flaps can also minimize the reperfusion injury even after the preconditioned skin flap, and we design an experiment to confirm the guess.

We had two goals in the experiment:

1. To confirm the usefulness of the post-conditioning of the flap in minimization the flap loss after the preconditioned skin flap;
2. To find out one useful post-conditioning strategy for the survival of the flap.

## Materials and Methods

Forty New Zealand White rabbits weighing 4-5 kg were acclimated to the animal housing facility for at least 48 hours. All animals were kept at room temperature and a 12-hour day/night lighting system. All animals were free to reach water and food. Intramuscular injections of LA 200 oxytetracycline 20 mg/kg per day were given the night before the procedure and once every 7 days thereafter. This study was carried out in strict accordance with the recommendations in the Guide for the Care and Use of Laboratory Animals of the National Institutes of Health. The protocol was approved by the Committee on the Ethics of Animal Experiments of Anzhen Hospital. All surgery was performed with all efforts to minimize suffering.

### The axial pattern skin flap

All these animals were divided into 5 groups (Control group, ischemia group, post-conditioning group，post-conditioning 5 minutes later group and post-conditioning 10minutes later group) of 8 rabbits each.

**Figure 1:**
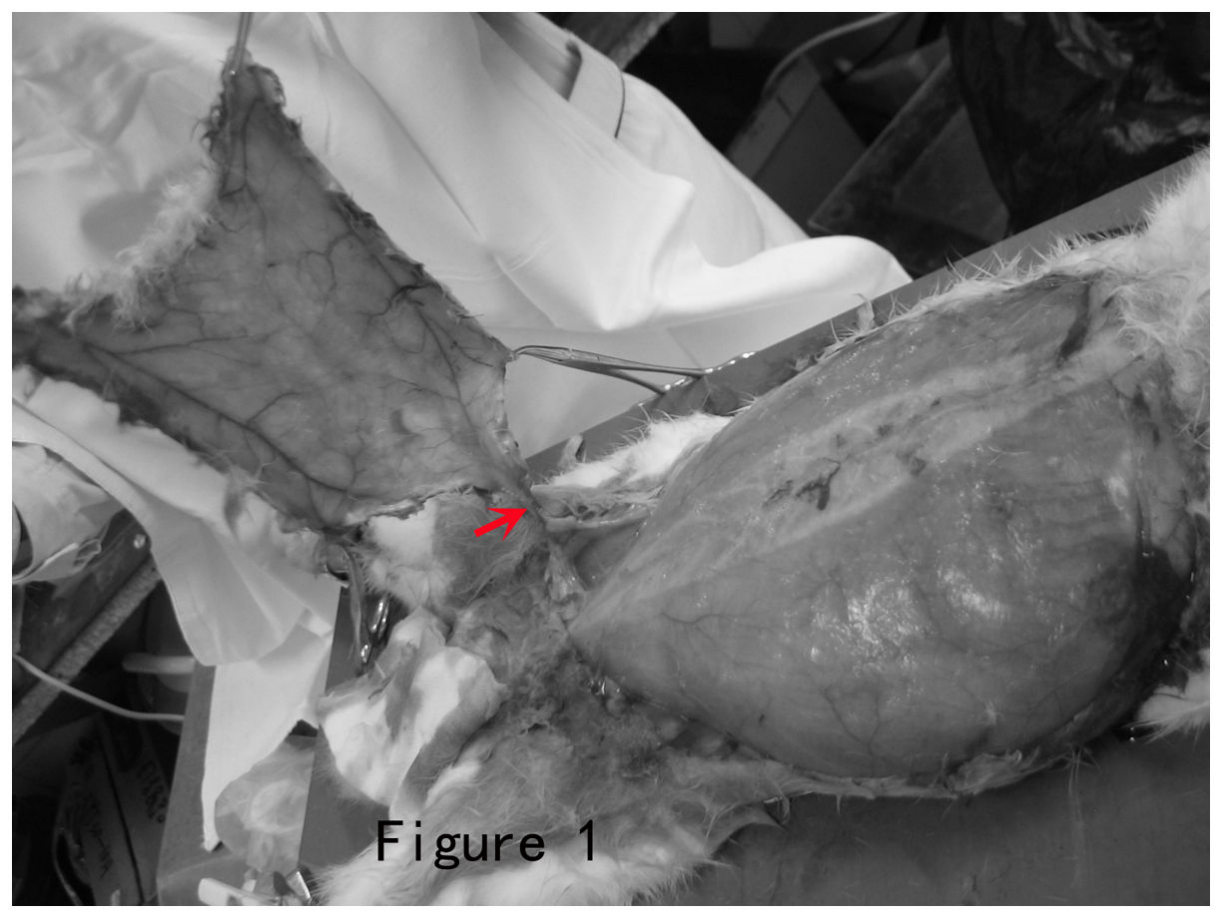
The overview of the elevation of the skin flap. The red arrow indicates the pedicle which is dissected.

Axial pattern island skin flaps were raised on the abdominal wall as Cederna [8] described. Abdominal fur was removed with clippers and depilatory cream. A 19 × 15 cm^2^ abdominal cutaneous island flap was elevated in rabbits. The flap consisted of skin, subcutaneous tissue, and panniculus carnosus. All perforators were divided. The only remaining attachment to the flap was the right superficial inferior epigastria pedicle. After flap elevation, a silicon sheet was placed on the muscle bed as a barrier to inhibit vascular invasion and sutured on the trauma surface with 7-0 polypropylene. Flaps were repositioned and sutured with 6-0 polypropylene sutures (Figure 1). The surviving area of the flap is assessed after 1 week.

Ischemia was induced by clamping the vessel(s) by using a microvascular clamp. The same V-type clamp was used both for the artery and the vein. During the ischemia procedure, because the blood into the flap remained still, so the flap was perfused with 3 ml heparin solution (5000 I.E. /ml) to avoid the blood coagulation of the skin flap. This solution is injected in the pedicle artery two times.

### Experiment protocol

The experiments were performed as Table 1.

**Table 1:** Surgical Procedure and Treatments Performance on the groups.

Groups	Treatment
1 Control(n=8)	No
2 Treatment 1(n=8)	Preconditioning
3 Treatment 2(n=8)	Preconditioning+Postconditioning
4 Treatment 3(n=8)	Preconditioning+Postconditioning 5 minutes later
5 Treatment 4(n=8)	Preconditioning+Postconditioning 10 minutes later

Group 1: Ischemia Group (8-Hours Ischemia) (n=8)

Flaps were prepared as described above and the vascular pedicles were occluded for 8h. At the end of 8 h of ischemia, they were anesthetized and the clamps removed.

Group 2: Pre-conditioning Group (8-Hours Ischemia - Pre-conditioning) (n=8)

The same procedure in group 1 was performed, but in addition, ischemic pre-conditioning was performed just before the ischemia. Ischemic pre-conditioning was applied by 2 periods of 15 minutes of ischemia/15 minutes of reperfusion cycle.

Group 3: Post-conditioning Group (Pre-conditioning -8-Hours Ischemia –Post-conditioning) (n=8)

The whole procedure was similar to group 2, inducing pre-conditioning with 2 periods of 15 minutes of ischemia/15 minutes of reperfusion cycle. But ischemic post-conditioning including 6 cycles of each 10s ischemia and 10s reperfusion (which lasted 2 minutes) was performed just after the induced ischemia.

Group 4: Post-conditioning 5 minutes later Group (Pre-conditioning -8-Hours Ischemia –Post-conditioning 5 minutes later) (n=8)

The whole procedure was similar to group 3, but ischemic post-conditioning was performed 5 minutes after the ischemia.

Group 5: Post-conditioning 10 minutes later Group (Pre-conditioning -8-Hours Ischemia –Post-conditioning 10 minutes later) (n=8)

The whole procedure was similar to group 3, including the method of post-conditioning, but ischemic post-conditioning was performed 10 minutes after the ischemia.

### Histologic Analysis

Biopsy specimens collected were prepared for histological examination as 4-mm-thick sections stained with hematoxylin and eosin. Tissue samples were taken from the midpoint which is 5mm distance from the medial border of the distal flap immediately before dissection. All sections were evaluated for neutrophil count under microscopy (100 magnification).

### Statistics

For all the animals which were divided into five groups, surviving and necrosis parts of the flaps were measured using a 2-dimensional planimetry in a blinded fashion 1 week after the flap was harvested. Surviving portions of the flaps were noted as a percentage of the whole flap area. One-way analysis of variance was used to compare the significance differences among different groups, and significance was set at P <0.05.

## Results

### Histologic Study

**Figure 2:**
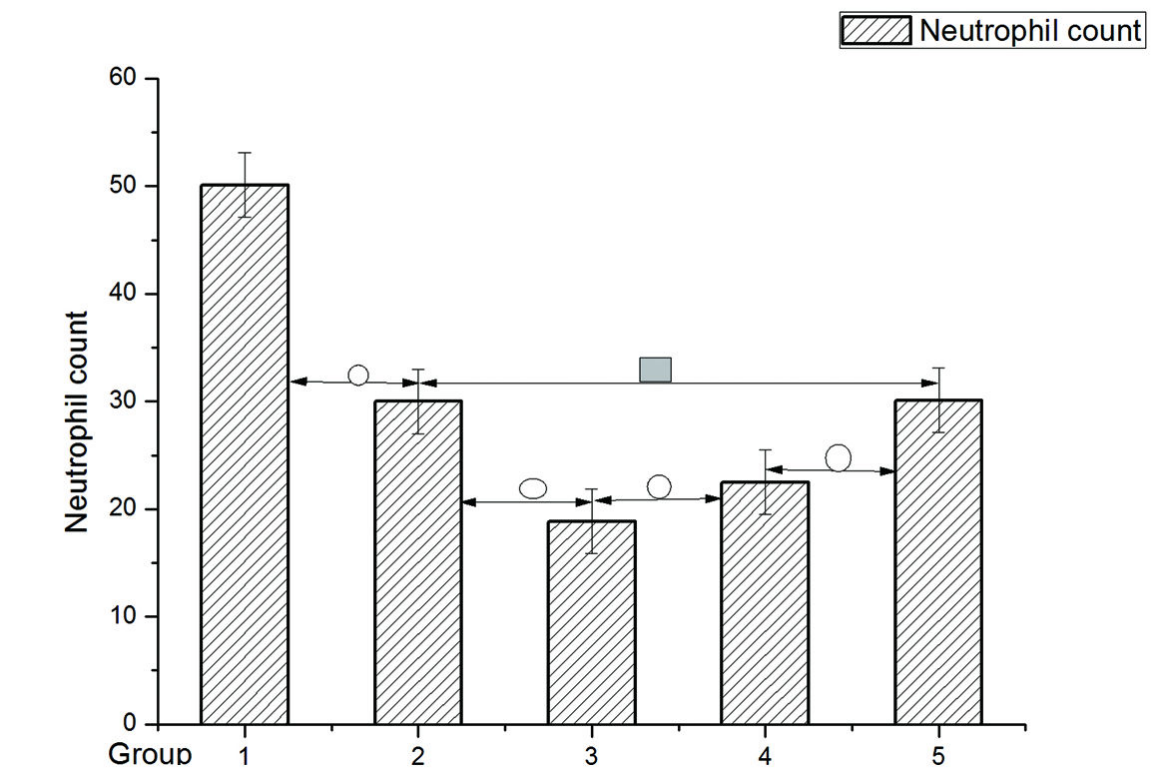
Neutrophil count among different groups. Data are expressed as mean. ±SD neutrophil count. **O** (p<0.05) compared between the neighboring groups. ■ (p>0.05) compares with 2/5 groups.

Among the varying groups, the neutrophil count varied: Group 1 was 50.12±5.91; Group 2, 30.00±2.00, and Group 3, 18.87±3; Group 4, 22.50±1.92; Group 5, 30.12±1.88.In addition, the LSD test showed significant difference (P <0.05) between the different groups except Group 2 to Group 5(See Figure 2 and Table 2).

**Table 2:** Neutrophil Count Occupied Among the Histologic Examination.

Animal No/ Group	1	2	3	4	5
1	48	30	23	23	29
2	51	32	22	25	30
3	38	33	13	19	27
4	49	27	18	22	29
5	56	28	16	21	32
6	57	29	21	22	33
7	53	30	15	24	31
8	49	31	23	24	30
Mean±SD	50.12±5.91	30.00±2.00	18.87±3.90	22.50±1.92	30.12±1.88

### Flap Survival

**Figure 3:**
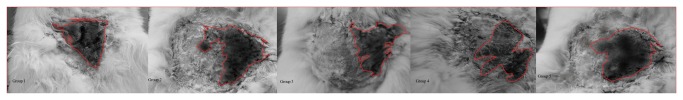
Comparison of the surviving portion of the skin flap. The red line indicates the necrosis part of the skin flap.

**Figure 4:**
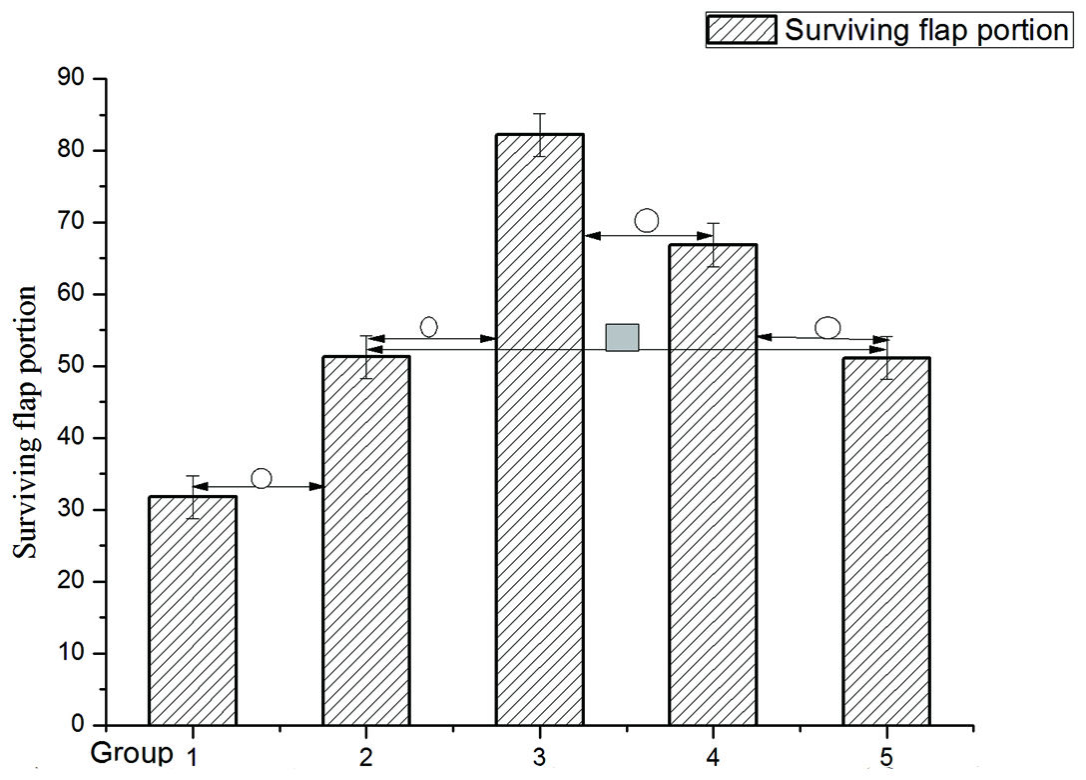
Neutrophil count among different groups. Data are expressed as mean ±SD neutrophil count. **O** (p<0.05) compared between the neighboring groups. ■ (p>0.05) compares with 2/5 groups.

Among the ischemia group, flap survival in Groups 1, 2, 3, 4 and 5 were 31.76±4.59, 51.26±3.24,82.18±5.28,66.85±3.87 and 51.13±2.90(See Figure 3). LSD test showed significant difference (P <0.05) between the different groups except Group 2 to Group 5 (See Figure 4 and Table 3).

**Table 3:** Surviving Flap Portion in Percentage 1 Week After the Surgical Procedure.

Animal No/ Group	1	2	3	4	5
1	32.10	45.10	75.10	61.80	55.20
2	25.90	50.90	80.50	70.50	47.30
3	39.20	51.20	76.20	61.00	52.20
4	25.20	49.20	88.20	65.50	46.80
5	30.50	50.50	87.30	70.40	53.650
6	34.10	55.10	79.50	70.70	50.30
7	32.60	54.50	82.40	68.50	52.10
8	34.50	53.60	88.30	66.40	51.50
Mean± SD	31.76±4.59	51.26±3.24	82.18±5.28	66.85±3.87	51.13±2.90

LSD (Least significance difference) test was used to compare the difference among group 1, 2, 3 ,4and 5.Stastitcally difference did not exist between group 2 and 5 ,but they all had difference when compared to group 1, group 3 or group 4.

We performed a Spearman correlation analysis showing an increase relation between neutrophil count and flap survival rate in the different groups (P <0.05).

## Discussion

During the last decade, microvascular anastomotic procedures have become well accepted in reconstructive surgery [9]. However, flap surgery is associated with surgical trauma and transfer-related ischemia-reperfusion injury, potentially resulting in flap failure with deleterious consequences for the patient [10]. It is accepted that reperfusion injury is an inflammatory process modulated by complex signaling mechanisms which ultimately leads to cell death. Restoration of blood flow is essential for any flap survival; however, such reperfusion can lead to IR injury via numerous inflammatory pathways [11].

The phenomenon of ischemic pre-conditioning (IPC) was first described in myocardium, where the brief periods of coronary artery occlusion and reperfusion followed by episode of sustained ischemia resulted in a significant reduction in the infarct size [12]. This finding was extrapolated to other tissues as it has been recently shown that ischemic pre-conditioning can also improve skin flap survival in animals [2,13].

Here the experiment was performed by clamping and releasing the clamps, which imitated the procedure of the anastomosis of the vessels of the free skin flap. We had demonstrated that ischemic post-conditioning could also improve skin flap survival in previous studies and the mechanism of the improvement is related to the inhibition of the inflammation of the skin flap [7,14]. But the effect of the ischemic post-conditioning on the pre-conditioning for the skin flap is not manifested. Here we devised an experiment to explore a useful strategy for the skin flap. The above experiments suggested that ischemic post-conditioning could also influence the preconditioned skin flap and improve the skin flap survival when post-conditioning just before the regain of the reperfusion 5 minutes earlier. We supposed this improvement might suggest that pre-conditioning or post-conditioning the skin flap suppress the inflammation through different pathway, so the post-conditioning of the preconditioned skin flap was related to further inhibition of inflammation of the skin flap.

There have been recent great strides in our understanding of the innate immune system, particularly in regard to the signaling mechanisms of Toll-like receptors (TLRs), whose primary role is the initial activation of immune cell responses. Mice that have been preconditioned displayed a pronounced reduction of TNF-α, iNOS, and COX-2 in the brains of wild-type TLR4 mice relative to TLR4-deficient mice [15]. TLRs are involved in the enhancement of cell damage following ischemia [16]. The activation of TLR signaling leads to ischemic preconditioning. Studies have shown TNF-α could activate neutrophils to release inflammatory mediators and play an important role in I/R injury. TNF-α also caused overexpression of adhesion molecules on both endothelial cells and leukocytes [17], and increased neutrophils aggregation and adhesion to endothelial cells Thus, inhibition of TNF-α production may prevent the subsequent neutrophils activation. Accumulating evidence indicates that ischemia alone may induce TNF-α mRNA [18]. While Guo JY suggested that protein and mRNA expression of TNF-α and ICAM-1 were markedly suppressed by IPO (Ischemic postconditioning) [19].

The suppression results of the inflammation induced either by pre-conditioning or post-conditioning can be added together. The inflammation is known to related to the no-reflow phenomenon, so this suppression can further reduce the occurrence of no-reflow.

The assessment technique for the neutrophil and surviving skin flap area was semi- quantitative, which would probably bias the final results, but we would introduce more precise technique for the assessment.

Something confused me is that why early versus 10 min-delayed post-conditioning had such a dramatic difference in flap survival. But if we attribute the protective effect of ischemic post-conditioning to its anti-inflammation role, then the variation might seem reasonable. The microvascular transfer of flaps is associated with an unavoidable surgical trauma and an ischemic period followed by microvascular reperfusion that can result in an inflammatory response in the respective tissues [20,21]. The recruitment of leukocytes in post-ischemic tissues is a hallmark of the inflammatory response [22]. Moreover, a series of inflammation reaction explode with a burst with the injury worsen. If the post-conditioning is performed early and the initial inflammation response is inhibited, the reperfusion injury of skin flap is minimized. While the ischemic post-conditioning is performed late, at first the amplified inflammation response is so heavy that the post-conditioning could not restrict it again; next the inflammatory mediators are widely released, and the inflammatory mediators might induce the formation of multi inflammation chain reaction on the contrary direction. So the reperfusion injury worsens and is beyond control.

Post-conditioning is feasible and practical when applied to humans in clinical situation. Surgeon can control the whole procedure of reperfusion under the elective (angioplasty, cardiac surgery, and transplantation) and emergency operation (replantation and vascular surgery). Moreover, the post-conditioning can apply to the ischemic tissue as an “after-injury” strategy easily. It is proved that ischemic post-conditioning has anti -inflammatory properties, which is of interest considering the significant role of inflammation in the hours and days following its involvement in adverse cardiac remodeling [23]. No reports proved that ischemic post-conditioning had any negative effects by now.

The benefits of the ischemic post-conditioning on the free skin flap have been proved due to its property of anti-inflammation, endothelial protection and so on. The addition of ischemic post-conditioning is used to enhance the protective effect of the pre-conditioning while causing little danger to the flap. All suggest the proposed strategy could be used potentially in future clinical practice.

## Conclusions

The usefulness of the ischemia post-conditioning plus ischemic pre-conditioning of skin flap may open up a therapeutic alternative in situations of unexpected and uncontrolled ischemic injury. Furthermore, a novel pharmacological strategy of skin flap protection may appear by studying the molecular mechanisms of conditioning.

## References

[1] Fan LK, Wang C, Hansen W, Welch WJ, Lee C (2000) Hsp72 induction: A potential molecular mediator of the delay phenomenon. Ann Plast Surg 44: 65–71. doi:10.1097/00000637-200044010-00011. PubMed: 10651368. 10651368

[2] Hosnuter M, Babucçu O, Kargi E, Altinyazar C (2003) Dual preconditioning: effects of pharmacological plus ischemic preconditioning on skin flap survival. Ann Plast Surg 50(4): 398–402. doi:10.1097/01.SAP.0000037261.84618.7F. PubMed: 12671383. 12671383

[3] Koenig WJ, Lohner RA, Perdrizet GA, Lohner ME, Schweitzer RT et al. (1992) mproving acute skin-flap survival through stress conditioning using heat shock and recovery. Plast Reconstr Surg 90(4): 659-664 10.1097/00006534-199210000-00016 1410003

[4] Matsumura H, Yoshizawa N, Vedder NB, Watanabe K (2001) Preconditioning of the distal portion of a rat random-pattern skin flap. Br J Plast Surg 54(1): 58–61. doi:10.1054/bjps.2000.3470. PubMed: 11121320. 11121320

[5] Salmi AM, Hong C, Futrell JW (1999) Preoperative cooling and warming of the donor site increase survival of skin flaps by the mechanism of ischaemic preconditioning: an experimental study in rats. Scand J Plast Reconstr Surg Hand Surg 33(2): 163–167. doi:10.1080/02844319950159406. PubMed: 10450572. 10450572

[6] Moon JG, Lim HC, Gye MR, Oh JS, Park JW (2008) Postconditioning attenuates ischemia-reperfusion injury in rat skin flap. Microsurgery. 28(7): 531-537. doi:10.1002/micr.20530. PubMed: 18683865. 18683865

[7] Huang L (2011) The impact of ischemia post-conditioning on the ischemia injury of skin flaps. Wounds. 23(11): 328-331.25881195

[8] Cederna PS, Chang P, Pittet-Cuenod BM, Razaboni RM, Cram AE (1997) The effect of the delay phenomenon on the vascularity of rabbit abdominal cutaneous island flaps. Plast Reconstr Surg 99(1): 183-193. doi:10.1097/00006534-199701000-00027. PubMed: 8982202. 8982202

[9] Urken ML, Weinberg H, Buchbinder D, Moscoso JF, Lawson W et al. (1994) Microvascular free flaps in head and neck reconstruction. Report of 200 cases and review of complications. Arch Otolaryngol Head Neck Surg 120: 633-640. doi:10.1001/archotol.1994.01880300047007. PubMed: 8198786. 8198786

[10] Benacquista T, Kasabian AK, Karp NS (1996) The fate of lower extremities with failed free flaps. Plast Reconstr Surg 98: 834-840. doi:10.1097/00006534-199610000-00013. PubMed: 8823023. 8823023

[11] Jassem W, Roake J (1998) The molecular and cellular basis of reperfusion injury following organ transplantation. Transplant Rev 12: 14. doi:10.1016/S0955-470X(98)80037-2.

[12] Murry CE, Jennings RB, Reimer KA (1986) Pre-conditioning with ischemia: A delay of lethal cell injury in ischemic myocardium. Circulation 74: 1124. doi:10.1161/01.CIR.74.5.1124. PubMed: 3769170. 3769170

[13] Matsumura H, Yoshizawa N, Vedder NB, Watanabe K (2001) Preconditioning of the distal portion of a rat random-pattern skin flap. Br J Plast Surg 54: 58-61. doi:10.1054/bjps.2000.3470. PubMed: 11121320. 11121320

[14] Huang L (2011) The impact of lidocaine on secondary ischemia injury of skin flaps. Transplant Proc 43(7): 2550-2553. doi:10.1016/j.transproceed.2011.04.018. 21911121

[15] Pradillo JM, Fernandez-Lopez D, Garcia-Yebenes I, Sobrado M, Hurtado O et al. (2009) Toll-like receptor 4 is involved in neuroprotection afforded by ischemic preconditioning. J Neurochem 109: 287–294. doi:10.1111/j.1471-4159.2009.05972.x. 19200341

[16] Cao CX, Yang QW, Lv FL, Cui J, Fu HB et al. (2007) Reduced cerebral ischemia-reperfusion injury in Toll-like receptor 4 deficient mice. Biochem Biophys Res Commun 353: 509–514. doi:10.1016/j.bbrc.2006.12.057. PubMed: 17188246. 17188246

[17] Issekutz TB (1990) Effects of six different cytokines on lymphocyte adherence to microvascular endothelium and in vivo lymphocyte migration in the rat. J Immunol 144: 2140–2146. PubMed: 2107253. 2107253

[18] Meldrum DR, Shenkar R, Sheridan BC, Cain BS, Abraham E et al. (1997) Hemorrhage activates myocardial NFkB and increases tumor necrosis factor in the heart. J Mol Cell Cardiol 29: 2849–2854. doi:10.1006/jmcc.1997.0506. 9344778

[19] Guo JY, Yang T, Sun XG, Zhou NY, Li FS et al. (, Oct 282011) Ischemic postconditioning attenuates liver warm ischemia-reperfusion injury through Akt-eNOS-NO-HIF pathway. J Biomed Sci, Oct 28;18: 79. doi:10.1186/1423-0127-18-79. PubMed: 22035453. 22035453 PMC3212808

[20] Kunkel EJ, Jung U, Bullard DC, Norman KE, Wolitzky BA et al. (1996) Absence of trauma-induced leukocyte rolling in mice deficient in both P-selectin and intercellular adhesion molecule 1. J Exp Med 183: 57-65. doi:10.1084/jem.183.1.57. PubMed: 8551244. 8551244 PMC2192429

[21] Eppihimer MJ, Granger DN (1997) Ischemia/reperfusioninduced leukocyte-endothelial interactions in postcapillary venules. Shock 8: 16-25. doi:10.1097/00024382-199707000-00004. PubMed: 9249908. 9249908

[22] Menger MD, Vollmar B (1996) Adhesion molecules as determinants of disease: from molecular biology to surgical research. Br J Surg 83: 588-601. doi:10.1002/bjs.1800830506. 8689199

[23] Thuny F, Lairez O, Roubille F, Mewton N, Rioufol G et al. (2012) Post-conditioning reduces infarct size and edema in patients with ST-segment elevation myocardial infarction. J Am Coll Cardiol 59: 2175–2181. doi:10.1016/j.jacc.2012.03.026. 22676937

