# Correction: What Happened if Various Kinds of Postconditioning Working on the Preconditioned Ischemic Skin Flaps

**DOI:** 10.1371/journal.pone.0122425

**Published:** 2015-03-30

**Authors:** The PLOS ONE Staff

The affiliation for author Lin Huang is incorrect. The correct affiliation is: Plastic and Reconstructive Surgery, Beijing Anzhen Hospital, Capital Medical Universtiy, Beijing, People’s Republic of China.
